# Comorbidity Between Mood Disorders and Chronic Somatic Diseases, With a Focus on Cardiometabolic Disease, and Its Mechanistic Crosstalk

**DOI:** 10.1155/da/7688691

**Published:** 2026-07-24

**Authors:** Hongzhen Du, Gangqiang Du, Qian Zhang, Yixuan Zhao, Jilin Fan, Yufeng Zhou, Zihao Sun, Chen Li, Wei Li

**Affiliations:** ^1^ School of Special Education and Rehabilitation, Binzhou Medical University, Yantai 264003, Shandong, China, bzmc.edu.cn; ^2^ First Clinical Medical College, Shandong University of Traditional Chinese Medicine, Jinan 250355, Shandong, China, sdutcm.edu.cn; ^3^ Department of Orthopedic Trauma, Binzhou Medical University Hospital, Binzhou 256603, Shandong, China, bzmc.edu.cn; ^4^ Department of Rehabilitation Medicine, Yantaishan Hospital, Yantai 264001, Shandong, China, ytsyy.com; ^5^ Department of Rehabilitation Medicine, Binzhou Medical University Hospital, Binzhou 256603, Shandong, China, bzmc.edu.cn; ^6^ Medical Research Center, Binzhou Medical University Hospital, Binzhou 256603, Shandong, China, bzmc.edu.cn

**Keywords:** cardiometabolic disease, chronic somatic diseases, comorbidity, gut-brain axis, mood disorders, sex differences

## Abstract

The comorbidity of mood disorders (MDs), represented by depression and anxiety, with chronic somatic diseases (CDs), particularly cardiometabolic conditions, poses a significant global public health challenge, markedly increasing the risk of adverse prognoses and all‐cause mortality. Epidemiological studies indicate that ~36% of patients with multimorbidity exhibit psychosomatic comorbidity, with higher risk populations concentrated among females, older adults, and socioeconomically disadvantaged individuals. This review systematically analyzes the epidemiological distribution patterns of MD–CD comorbidity, with the strongest emphasis on cardiometabolic diseases—diabetes, metabolic syndrome (MetS), obesity, hypertension (HTA), and cardiovascular disease (CVD)—for which the mechanistic data are most developed, while also addressing osteoarthritis, psoriasis, inflammatory bowel disease (IBD), cancer, and neurological disorders. By integrating molecular mechanisms with clinical evidence, we highlight core pathological pathways and critically evaluate the strength of evidence supporting each: hypothalamic–pituitary–adrenal (HPA) axis dysfunction and insulin resistance (IR), substantiated by clinical data and Mendelian randomization; NLRP3 inflammasome‐mediated neuroinflammation as a transdiagnostic inflammatory node; gut–brain axis dysregulation involving bile acid–GLP‐1 signaling and gut‐derived metabolites such as trimethylamine N‐oxide (TMAO); and the kynurenine pathway (KP) as a branched metabolite axis with organ‐specific consequences. Sex‐specific mechanisms, particularly estrogen‐regulated neuroendocrine and metabolic pathways, are identified as consistent biological modifiers of this comorbidity. A bidirectional vicious cycle underpins MD–CD comorbidity: metabolic abnormalities exacerbate limbic system dysfunction via HPA‐axis hyperactivity, neuroinflammatory cascades, and disrupted gut–brain signaling, while MDs aggravate metabolic dysregulation through neuroendocrine disturbances and reduced treatment adherence.

## 1. Introduction

Mood disorders (MDs) represent a group of mental illnesses primarily characterized by significant dysregulation of emotional regulation [1]. Globally, ~7.2% of the population is affected by MD, while 4% suffers from anxiety, with incidence rates rising markedly with age [2]. During the COVID‐19 pandemic, the prevalence of anxiety and depression increased by 26% and 28%, respectively, establishing MD as one of the leading causes of disability and reduced healthy life expectancy in recent years [3]. Notably, MD frequently coexists with one or more chronic somatic diseases (CDs) in a psychosomatic comorbidity pattern rather than occurring in isolation [4–6].

CD, defined as noncommunicable conditions lasting over 3 months and affecting physiological systems, encompass metabolic disorders, cardiovascular diseases (CVDs), autoimmune diseases, MD, and musculoskeletal disorders, among others. According to the 2021 Global Burden of Disease Study, CD accounted for 60.1% of the global disease burden [3]. MD–CD comorbidity is strongly associated with poor clinical outcomes [7], contributing to elevated all‐cause mortality and increased public health burdens [8, 9]. Populations with comorbid depression exhibit 23%–85% higher risks of type 2 diabetes (T2D), CVD, and multimorbidity compared to those without [10], while those with CD demonstrate heightened susceptibility to MD [11].

Shared pathogenic mechanisms underlie MD–CD comorbidity, extending across the full cardiometabolic spectrum. Dysregulation of the hypothalamic–pituitary–adrenal (HPA) axis, leading to aberrant glucocorticoid secretion, not only induces insulin resistance (IR) but also suppresses hippocampal neurogenesis to promote depression—an association documented not only in diabetes but also in metabolic syndrome (MetS), obesity, hypertension (HTA), and CVD [12–14]. IR itself constitutes a transdiagnostic mechanistic bridge whose endothelial, neuroinflammatory, and neurotrophic consequences converge to elevate the MD risk across the cardiometabolic spectrum [15–17]. In chronic inflammatory states, the NLRP3 inflammasome serves as a shared inflammatory node linking cardiometabolic disease and MD [18], while angiopoietin‐like protein 8 (ANGPTL8)‐enriched exosomes derived from adipose tissue trigger hippocampal synaptic damage via the PirB signaling pathway, disrupting prefrontal‐limbic neural circuitry [18–20]. Within the gut–brain axis, dysregulation of the bile acid–TGR5/cAMP pathway and impaired glucagon‐like peptide‐1 (GLP‐1) signaling collectively contribute to serotonergic neurotransmission deficits [21, 22], and gut‐derived metabolites—including trimethylamine N‐oxide (TMAO) and indole‐3‐propionic acid (IPA)—operate across a gut–brain–heart axis to sustain the bidirectional comorbidity. Peripheral inflammation further diverts tryptophan toward the kynurenine pathway (KP), a branched metabolite axis whose organ‐specific outputs differ between cardiovascular and neuropsychiatric consequences [23]. Sex‐specific mechanisms, particularly estrogen‐regulated modulation of HPA‐axis reactivity, serotonergic neurotransmission, and neuroinflammation, consistently modify these shared pathways, with the causal pathway from depression to cardiometabolic disease being more pronounced in females. The mechanistic pathways summarized here span a wide spectrum of evidence: HPA‐axis hyperactivity and IR are substantiated by human clinical data and Mendelian randomization studies [24], whereas the roles of ANGPTL8/PirB signaling, specific NLRP3‐mediated neuroinflammatory circuits, and the bile acid–TGR5/cAMP cascade are established primarily in rodent models and require translational validation. This comorbid relationship exhibits bidirectional vicious cycle characteristics.

**Figure 1:**
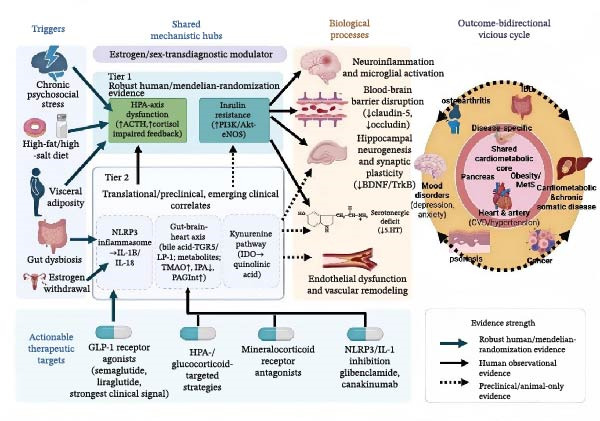
An evidence‐stratified framework linking mood disorders and cardiometabolic/chronic somatic disease. 5‐HT, 5‐hydroxytryptamine; ACTH, adrenocorticotropic hormone; BDNF, brain‐derived neurotrophic factor; CVD, cardiovascular disease; eNOS, endothelial nitric oxide synthase; GLP‐1, glucagon‐like peptide‐1; HPA, hypothalamic–pituitary–adrenal axis; IBD, inflammatory bowel disease; IDO, indoleamine‐2,3‐dioxygenase; IL‐1β, interleukin‐1 beta; IL‐18, interleukin‐18; IPA, indole‐3‐propionic acid; MetS, metabolic syndrome; NLRP3, NLR family pyrin domain‐containing protein 3; PAGIN, platelet‐activating factor acetylhydrolase inhibitory protein; PI3K, phosphoinositide 3‐kinase; T2D, type 2 diabetes; TGR5, Takeda G‐protein‐coupled receptor 5; TMAO, trimethylamine N‐oxide; TrkB, tropomyosin receptor kinase B.

This review systematically delineates the epidemiological features of MD comorbidity with metabolic diseases, CVD, and immune disorders; elucidates multisystem interactive mechanisms involving HPA‐axis dysfunction, IR, NLRP3‐mediated neuroinflammation, gut–brain axis dysregulation, and the KP; examines sex‐specific mechanisms that modify these shared pathways; and explores the translational potential of emerging therapeutic strategies targeting GLP‐1 signaling, PPARγ/β‐catenin signaling, and CD19+ B‐cell modulation. Reflecting the distribution of the available evidence, the review is organized as a progression from epidemiology to shared biology, then to sex‐specific mechanisms, disease‐specific examples, and finally to treatment implications; it is deliberately weighted toward cardiometabolic conditions, namely, diabetes, MetS, obesity, HTA, and CVD, for which the mechanistic data are most developed, whereas osteoarthritis, psoriasis, inflammatory bowel disease (IBD), cancer, and neurological disorders are discussed more briefly. Throughout, we critically evaluate the strength of evidence supporting each pathway—distinguishing robust clinical and Mendelian randomization data from translational animal findings and emerging preclinical hypotheses—to identify the most actionable routes from biology to treatment. Figure 1 presents an evidence‐stratified mechanistic framework.

## 2. Epidemiological Characteristics of MD in Comorbidity

Current research highlights that the comorbidity of MD exhibits multidimensional biopsychosocial regulatory features, with bidirectional pathological interactions. Epidemiological data reveal that 8.3% of all patients and 36.0% of those with multimorbidity experience psychosomatic comorbidity, with higher rates observed in females, older adults, and individuals in socioeconomically disadvantaged regions, where prevalence nearly doubles compared to affluent areas [25]. Such comorbidities are nonrandom, displaying significant genetic correlations and geographical heterogeneity [26, 27]. For instance, the comorbidity rate between diabetes mellitus (DM), used hereafter as an umbrella term for DM encompassing both type 1 (T1D) and type 2 (T2D) diabetes, with T2D reserved for findings confined to T2D, and mental disorders markedly exceeds that in the general population. Canadian studies report a 7.9% prevalence of MD in patients with T1D, while data from developing countries indicate that over 30% of diabetes patients exhibit depressive symptoms. In developed nations, T2D patients show pronounced gender disparities in depression incidence, with U.S. data revealing rates of 15.4% in females vs. 9.1% in males.

The bidirectional metabolic‐MD association is particularly prominent in major depressive disorder (MDD)‐T2D comorbidity. Patients with MetS face a 23% increased risk of MDD compared to the general population [28]. In China, the prevalence of MDD among T2D patients reached 12.8% [29], which is significantly higher than the 3.0%–7.5% observed in European populations. Conversely, patients with MDD exhibit an 8.7%–18.9% elevated risk of developing T2D. This comorbidity demonstrates vulnerability during critical developmental periods: U.S. hospitalization data indicate a 78.9% MDD comorbidity rate among adolescents with MetS, far exceeding depression and anxiety rates in adults [30]. Aging further amplifies this risk, particularly in individuals >55 years old [31]. Among MetS components, central obesity, HTA, and hyperlipidemia show the strongest associations with depressive symptoms [32]. Compared to non‐T2D individuals, T1D patients exhibit significantly higher depression rates, while T2D patients demonstrate elevated prevalence [33]. Specifically, depression affects 14.35% of T1D patients, with anxiety disorders present in 12.56% [34]. MDD similarly increases the T2D risk by 22% [35]. Depressive symptoms correlate strongly with IR and inflammatory markers in T2D patients, with somatic symptoms playing a dominant role in these associations [36].

The bidirectional obesity–depression relationship displays notable population heterogeneity. In the United States, 53% of individuals with MD are obese, whereas German data indicate depression risks in only 2.2% of males and 3.7% of females who are obese [37–39]. Among treatment‐resistant patients with MDD, the MetS prevalence reaches 38%, with lipid metabolism dysregulation frequently observed, suggesting that lipid abnormalities may exacerbate treatment resistance by altering BBB permeability and destabilizing neuronal membranes [40, 41].

CVD‐MD associations are also evident. Patients with HTA exhibit depression/anxiety rates of 17.9%–47%, with treated individuals showing a higher depression prevalence than untreated counterparts [42–44]. Post‐stroke depression incidence exceeds that of coronary heart disease‐related depression, implicating direct cerebrovascular damage as a stronger driver of affective disorders compared to myocardial ischemia [45].

Gender disparities in psychosomatic comorbidity emerge in osteoarthritis, with depression/anxiety rates of 15.4%–29% in males and 21.8%–34% in females [46, 47]. Psoriasis patients demonstrate depression and anxiety rates of 19% and 36%, respectively, while IBD (Crohn’s disease and ulcerative colitis) significantly elevates the depression risk [48]. In Africa, depression and anxiety prevalence among patients with cancer exceeds 50%, with Lebanese cancer patients similarly showing depression rates above 50%—markedly higher than those in the global average. These disparities may stem from delayed diagnoses, inadequate palliative care, and culturally amplified stigma [49–52]. Table 1 summarizes the incidence rates of the relevant comorbidities.

**Table 1:** Epidemiology of comorbidity between mood disorders and chronic somatic diseases.

Comorbidity (primary diseases/secondary diseases)		Prevalence	Data source	Target population	References
T1D	Mood disorders	7.9% (95% CI: 3.1–12.7)	Canadian Community Health Survey; Canadians	—	[53]
T1D and T2D	Depression	12.4% (95% CI: 11.0%–13.8%); female (15.4%) male (9.1%)	National Ambulatory Medical Care Survey 2014–2019; United States	62.7 years	[30]
T1D and T2D	Depressive symptoms	T1D (30.7%) T2D (33.1%)	The International Diabetes Management Practices Study; Developing Countries	—	[54]
T2D	MDD	12.8%	The International Prevalence and Treatment of Diabetes and Depression; China	53.5 years	[29]
T2D	MDD	3.0%	The Scottish Diabetes Research Network – National Diabetes Dataset (United Kingdom)	58.9 years	[55]
T2D	MDD	5%; Female (95% CI: 0.69–2.04), Male (95% CI: 1.02–2.75)	The Maastricht Study; Netherlands	—	[56]
T2D	MDD	7.5%	The UK Biobank; United Kingdom	Median age: 59.0 years	[57]
MDD	T2D	8.7% (95% CI: 7.3–10.2%)	—	—	[58]
MetS	Depression Anxiety	26.6% 21.8%	Mental Health Service Drenthe; Netherlands	46.0 years	[59]
Obesity	Depression	Male: 2.2% (95% CI: 0.8–2.0) Female: 3.7% (95% CI: 1.3–3.0)	The nationwide German health interview and examination survey; German	—	[37]
MDD	Overweight/obesity	40%	Dire Dawa Psychiatric Center; Eastern Ethiopia	37.18 ± 12.59 years	[38]
MDD	Overweight/obesity	35.2%	The Lucio Bini Psychiatric Center; Cagliari	48.5 years	[39]
Obesity	Depression	7.8%	Korea National Health and Nutrition Examination Survey; Republic of Korea	Female; 49.1 years	[40]
Depressive	Obesity	53%	The RAINBOW clinical trial; United States	—	[41]
MDD	Overweight Obesity	56.00% 3.73%	The First Hospital of Shanxi Medical University; China	34.0 years	[60]
MDD	Overweight Obesity	30.9% 21.2%	Early Medication Change (EMC) trial; Germany	42.0 years	[61]
Obesity	Mood disorders Anxiety	25.9% 18.7%	Canadian Community Health Survey; Canada	—	[62]
HTA	Depression	47% (treated patients with hypertension) 33% (untreated patients with hypertension)	The MONICA/KORA study data; Germany	—	[42]
HTA	Depression Anxiety	28.5% 21.6%	China	Female; 56.4 years	[44]
HTA	Depression	17.9% (95% CI: 13.0%–23.4%)	EMBASE, African Index Medicus and African Journals OnLine; Africa	—	[43]
HTA	Post‐stroke depression	32%	United States	61.7 ± 11.1 years	[45]
HTA	Depression	17.4%	World Mental Health; Colombia	53.56 ± 10.28 years	[63]
MDD	HTA	18.9%	Europe	50.3 ± 14.1 years	[64]
MDD	HL	48%	The Hospital for Sick Children; Canada	15.04 ± 1.81 years	[65]
Treatment‐resistant depression	MetS	38%	FondaMental Advanced Centers of Expertise in Resistant Depression (France)	—	[66]
MDD	HL TC TG LDL‐C HDL‐C	72.17% 30.17% 57.39% 14.68% 6.52%	Wuhan Mental Health Center; China	18–60 years	[67]
MetS	MDD	78.9%	National inpatient sample; United States	16.3 ± 1.7 years	[68]
OA	Depression	Male: 15.4% Female: 21.8%	The 2010–2012 Korea National Health and Nutrition Examination Survey; Korea	Male: 58.1 years Female: 56.9 years	[46]
OA	Depression	34% (MDD19.9%)	Western Health; Australia	54.1 ± 15.7 years	[47]
OA	Anxiety	28.06%	The Second Hospital of Shanxi Medical University; China	64.02 years	[48]
OA	Depression Anxiety	29% 22%	The Rush University Medical Center; United States	66.6 ± 11.7 years	[69]
OA	Depression Anxiety	30% (95% CI: 18.0%–43%) 27% (95% CI: 24%–30%)	—	—	[70]
Cancer	Depression Anxiety	53.21% (95% CI: 47.47%−58.94%) 53.32% (95% CI: 46.85%−59.80%)	African	—	[71]
Cancer	Depression Anxiety	63.4% 50.5%	American University of Beirut Medical Center; Lebanon	55.73 years	[72]
Coronary heart disease	Depression	10%	The Tilburg Health Outcomes Registry of Emotional Stress after Coronary Intervention; Netherlands	64.7 years	[52]
Stroke	MDD Anxiety	17.7% (95% CI: 15.6%−20.0%) 9.8% (95% CI: 5.9%−14.8%)	—	65 years	[49]
Crohn’s disease Ulcerative colitis	Depression Anxiety Depression Anxiety	17.5% 3.5% 14.2% 3.0%	Royal College of General Practitioners Research and Surveillance Centre; United Kingdom	—	[50]
Psoriasis	Depression Anxiety	19% 36%	Hospital das Clínicas of the Faculdade de Medicina de Botucatu; Brazil	52.1 ± 13.8 years	[51]

Abbreviations: HDL‐C, high‐density lipoprotein cholesterol; HL, hyperlipidemia; HTA, hypertension; LDL‐C, low‐density lipoprotein cholesterol; MDD, major depressive disorder; MetS, metabolic syndrome; OA, osteoarthritis; T1D, type 1 diabetes; T2D, type 2 diabetes; TC, total cholesterol; TG, triglycerides.

## 3. Shared Mechanisms Underlying MD–CD Comorbidity

### 3.1. HPA Axis Dysfunction

The HPA axis, the body’s primary stress response system, regulates adrenal glucocorticoid secretion to mediate adaptation to external stressors [73, 74]. Its dysfunction plays a pivotal role in both MD and MetS, particularly in diabetes patients with comorbid MD [75]. HPA‐axis dysregulation is a hallmark of diabetes, characterized by elevated circulating adrenocorticotropic hormone (ACTH) and cortisol levels [12, 13], leading to hyperactivation and impaired negative feedback. Genetic studies in patients with diabetes reveal that mutations in corticotropin‐releasing hormone receptor 2 (CRHR2) and glucocorticoid‐receptor (NR3C1) genes induce HPA‐axis overactivation and excessive cortisol secretion, disrupting glucose homeostasis and driving T2D‐MD comorbidity [76–78]. Melanocortin receptor genes, including MC1R, MC3R, and MC4R, influence lipid metabolism and IR through interactions with ACTH, whereas MC5R exacerbates comorbidity by modulating inflammatory and immune responses [79]. HPA‐axis dysfunction also triggers chronic cytokine‐mediated inflammation, activating the NLRP3 inflammasome to promote depression. Notably, the anti‐diabetes drug glibenclamide inhibits NLRP3 inflammasome activation, ameliorating depression–diabetes comorbidity [18].

The hippocampus is critical in the stress response. Stress‐induced cortisol via the HPA‐axis acts on hippocampal mineralocorticoid and glucocorticoid receptors, upregulating NR3C1, Nurr1, and interleukin (IL)‐1β protein expression [14, 80]. NR3C1 activates SGK3, inducing autophagic death of hippocampal neural stem cells and suppressing neurogenesis, thereby promoting depression [81, 82]. The hippocampus normally restores HPA‐axis function via hippocampal‐hypothalamic feedback; disruption of this mechanism exacerbates stress‐related IR. Selective serotonin reuptake inhibitors downregulate HPA‐axis activity and reduce hippocampal mineralocorticoid‐receptor (MR) expression, alleviating depression while reversing T2D‐associated IR [83].

Beyond diabetes, HPA‐axis dysregulation is similarly implicated across the broader cardiometabolic cluster comorbid with MD, including MetS, obesity, HTA, and CVD, though the depth of human evidence varies by condition. In MetS, a nested case–control study drawn from the Whitehall II cohort found that individuals meeting MetS criteria had significantly higher 24‐h urinary cortisol metabolite and normetanephrine outputs than matched controls, directly implicating combined HPA‐axis and sympathoadrenal hyperactivity in human MetS pathophysiology [84]. This relationship is not merely cross‐sectional: a 14‐year prospective analysis of over 10,000 Whitehall II civil servants demonstrated a dose–response relationship between cumulative chronic work stress and incident MetS, independent of baseline obesity, supporting stress‐driven HPA activation as an antecedent rather than a consequence of MetS [85]. The intersection with MDs was directly tested in the InCHIANTI study, where depressive symptoms and 24‐h urinary cortisol independently predicted MetS, and a significant interaction was observed between depressed mood and cortisol output, such that individuals with both hypercortisolemia and depression had nearly double the odds of MetS compared with those with neither, suggesting a distinct “hypercortisolemic depression” phenotype [86]. Genetic evidence parallels these clinical findings: in a Brazilian Mennonite population, homozygosity for the NR3C1 promoter variant rs10482605 ^∗^C and several glucocorticoid‐receptor haplotypes were independently associated with several‐fold increased MetS susceptibility, mirroring the same NR3C1 locus implicated in T2D‐MD comorbidity and suggesting glucocorticoid‐receptor sensitivity as a shared genetic substrate across cardiometabolic phenotypes [87]. For obesity specifically, a complementary local mechanism operates independently of circulating cortisol: human adipose biopsy studies show that 11β‐hydroxysteroid dehydrogenase type 1, the enzyme that regenerates active cortisol from inert cortisone, is selectively upregulated in the subcutaneous adipose tissue of obese individuals and correlates with body mass index and IR even when systemic cortisol is normal, indicating that obesity can drive a state of tissue‐specific glucocorticoid excess that circulating cortisol measurements alone would miss [88].

In HTA, the most rigorous causal evidence to date comes from a two‐sample Mendelian randomization study using SERPINA6/SERPINA1 cortisol‐associated genetic variants, which found that genetically predicted plasma cortisol was robustly associated with increased HTA risk and higher systolic and diastolic blood pressure. Notably, this same analysis found no genetic evidence for a causal effect of cortisol on depression, T2D, or osteoporosis, and the HTA association was substantially attenuated after adjusting for waist circumference, indicating that central, obesity‐mediated glucocorticoid excess rather than a direct cortisol‐mood pathway most plausibly drives this limb of the comorbidity [89]. This finding is important for interpreting the broader HPA‐MD literature as it demonstrates that cortisol’s causal cardiometabolic effects and its associations with mood are not interchangeable and should be evaluated as distinct hypotheses. At the epidemiological level, the directionality also appears asymmetric: a meta‐analysis of prospective cohort studies found that depression significantly increased subsequent HTA incidence [90], whereas a separate meta‐analysis restricted to prospective cohorts in older adults found that baseline HTA did not significantly predict subsequent depression (pooled relative risk 1.16, 95% CI 0.91–1.42) [91], a pattern that, together with the Mendelian randomization findings above, suggests that depression may more often precede than follow HTA in this particular limb of the comorbidity.

In CVD specifically, the causal evidence is more contested than that in HTA. Using prospective cohort data from Whitehall II and InCHIANTI together with one‐ and two‐sample Mendelian randomization, one analysis reported that flatter diurnal cortisol slopes and elevated 24‐h urinary cortisol predicted incident cardiovascular mortality, with genetic instruments for morning cortisol yielding a directionally concordant, though not independently statistically significant, association with incident coronary heart disease (OR 1.06, 95% CI 0.98–1.15) across more than 120,000 cases [92]. By contrast, a separate bidirectional Mendelian randomization analysis using cortisol instruments from the CORtisol NETwork (CORNET) consortium found no evidence that genetically predicted cortisol was associated with ischemic heart disease, leading the authors to conclude that their findings cast doubt on a direct cortisol‐driven causal pathway to cardiovascular risk [93]. This discordance between two methodologically similar Mendelian randomization studies indicates that, unlike the cortisol–HTA link described above, the causal role of circulating cortisol in CVD specifically remains unresolved. The mood‐disorder side of this pathway has firmer support: genetic liability to depression was shown, via Mendelian randomization, to causally increase the risk of coronary artery disease (OR 1.14, 95% CI 1.06–1.24) and myocardial infarction (OR 1.21, 95% CI 1.11–1.33), with T2D and smoking acting as significant mediators [94], a directional finding that parallels the depression‐to‐T2D causal estimate described above [24] and positions depression as a plausible upstream contributor to cardiovascular pathology even where the reverse, cortisol‐mediated pathway remains genetically unconfirmed. At the level of individual susceptibility genes, polymorphisms in FKBP5, a cochaperone protein that regulates glucocorticoid‐receptor sensitivity and HPA‐axis negative feedback, were associated with susceptibility to comorbid depression in a Northern Chinese cohort of patients with coronary artery disease. Because FKBP5 has separately been linked to IR and obesity, this gene illustrates how a single HPA‐axis regulatory node may plausibly extend across multiple cardiometabolic disease categories, though this remains a single‐cohort candidate‐gene finding awaiting replication [95].

Taken together, the genetic and clinical evidence supporting HPA‐axis involvement in T2D‐MD comorbidity varies considerably in strength. The associations of NR3C1, CRHR2, and melanocortin receptor (MC1R–MC5R) variants with this comorbidity rest primarily on familial linkage and candidate‐gene analyses conducted in relatively small, ethnically homogeneous Italian pedigrees, and the original reports themselves explicitly call for replication in large, independent, and ancestry‐diverse cohorts before these variants can be considered established risk factors [76, 77]. By contrast, elevated circulating ACTH and cortisol with impaired glucocorticoid negative feedback in patients with T2D has been documented across multiple independent clinical studies using dexamethasone‐suppression tests and 24‐h urinary free cortisol measurement, placing HPA‐axis dysregulation on substantially firmer clinical footing [12, 13]. At the causal‐inference level, a two‐sample bidirectional Mendelian randomization study by Maina et al. found a significant causal effect of depression on T2D risk (OR = 1.26; 95% CI 1.11–1.44) but no evidence for the reverse direction, indicating that depression is more likely to precede and drive T2D development than vice versa [24]. This evidence‐tiering extends beyond diabetes to the wider cardiometabolic cluster. Clinical and prospective cohort evidence linking HPA‐axis/cortisol dysfunction to MetS is robust and replicated across independent cohorts [84–86], and the causal direction from cortisol to HTA is further corroborated by Mendelian randomization, whereas the same analysis explicitly found no causal cortisol‐to‐depression effect, cautioning against assuming a single bidirectional cortisol‐mood pathway across all cardiometabolic phenotypes [89]. For CVD, Mendelian randomization evidence for a cortisol‐driven causal pathway is conflicting, with one analysis directionally consistent and another null [92, 93], while a separate Mendelian randomization analysis indicates that depression itself causally elevates coronary artery disease and myocardial infarction risk [94], mirroring the depression‐to‐T2D causal direction identified by Maina et al. The NR3C1‐MetS and FKBP5‐CAD genetic associations described above, like the candidate‐gene findings for T2D, derive from single cohorts or small samples and require replication in larger, ancestry‐diverse populations before they can be considered established risk loci [87, 95].

### 3.2. IR

IR, a core feature of diabetes, is also a key factor and predictor in MD pathogenesis [15–17, 96]. Insulin signaling is widely distributed in the central nervous system, particularly in emotion‐ and cognition‐related regions like the hippocampus, prefrontal cortex, and hypothalamus. IR impairs neuronal glucose uptake, disrupts synaptic plasticity, and weakens cognitive and emotional regulation [97–99]. It upregulates monoamine oxidase A/B (MAO‐A/B) expression in astrocytes, accelerating dopamine degradation and clearance, which induces anxiety‐ and depression‐like behaviors [100]. An astrocyte‐specific insulin receptor knockout replicates these effects [101]. IR damages the BBB via oxidative stress, endothelial signaling interference, and tight‐junction protein disruption [102, 103]. The association between peripheral IR and depressive symptoms is supported by human prospective cohort studies and meta‐analyses [15–17], placing central IR on solid clinical footing. However, the specific molecular pathways described above—including Adora2a‐driven BBB tight‐junction loss, astrocyte‐specific insulin receptor signaling, BH4 oxidation, and the reversing effects of recombinant FGF21 or HDAC3‐selective inhibitors—are defined primarily in rodent models and in vitro preparations. Their relative contribution in humans remains to be established in prospective translational studies. Diet‐induced IR increases endothelial adenosine receptor 2a (Adora2a) expression. Adora2a activation leads to the loss of BBB tight‐junction proteins, specifically claudin‐5 and occludin [104, 105]; it upregulates endothelial inflammatory markers, including ICAM‐1 and E‐selectin [106, 107] and promotes immune molecule leakage, aggravating IR‐induced central nervous system damage and impairing synaptic plasticity and hippocampal‐dependent cognition [108]. Tetrahydrobiopterin (BH4), a cofactor for endothelial nitric oxide synthase, mitigates Nox2‐driven oxidative stress and enhances NO production [109]. Hyperinsulinemia oxidizes BH4, exacerbating BBB endothelial dysfunction [110, 111]. Nrf2 upregulates antioxidant enzymes such as HO‐1 and catalase (CAT) to counteract IR‐induced oxidative stress, but histone deacetylase 3 suppresses this process [112]. Recombinant FGF21 and HDAC3‐selective inhibitors reverse these effects [113, 114].

Beyond diabetes, IR constitutes a mechanistic bridge linking the broader cardiometabolic cluster, including obesity, HTA, and CVD, to MD, operating through a shared vascular and neuroinflammatory axis that extends the pathways described above. In the vasculature, IR selectively impairs the PI3K/Akt branch of insulin signaling in endothelial cells, reducing eNOS‐mediated NO production while leaving the MAPK branch intact, thereby promoting compensatory endothelin‐1 secretion, endothelial dysfunction, and arterial stiffness [115, 116]. This pathway‐selective endothelial IR is shared across obesity, HTA, and atherosclerosis and converges on the same vascular mechanisms that impair cerebral perfusion and BBB integrity in depression [117]. Mendelian randomization using 53 SNPs for the IR phenotype from a genome‐wide association study of up to 188,577 participants confirmed a causal effect of genetically predicted IR on HTA and venous thromboembolism, with high‐density lipoprotein cholesterol (HDL‐C) and triglycerides partially mediating the HTA association [118], providing genetic‐level evidence that IR‐driven cardiometabolic risk is not confined to the diabetic state. In obesity, central leptin resistance codevelops with IR and synergistically amplifies mood dysregulation: leptin normally supports hippocampal synaptic plasticity and serum brain‐derived neurotrophic factor (BDNF) expression via the JAK2/STAT3 and PI3K/Akt/mTOR pathways, but obesity‐induced leptin resistance impairs these neurotrophic signals, reducing hippocampal neurogenesis and worsening depressive vulnerability [119]. A large meta‐analysis further confirmed that fasting insulin and homeostatic model assessment of insulin resistance (HOMA‐IR) are significantly elevated in patients with MDD relative to controls, independent of BMI, establishing hyperinsulinemia per se, rather than obesity alone, as an independent correlate of depressive pathology [120]. Collectively, these findings reframe IR not as a diabetes‐specific mediator but as a transdiagnostic cardiometabolic mechanism whose endothelial, neuroinflammatory, and neurotrophic consequences converge to elevate the MD risk across the full spectrum of cardiometabolic disease. The human evidence is strongest for IR as a correlate and prospective predictor of depressive symptoms; the specific vascular and leptin‐mediated neurobiological pathways described here remain primarily defined in animal and in vitro models and require translational confirmation.

### 3.3. Gut–Brain Axis

The gut–brain axis is a critical hub for diabetes‐MD comorbidity, modulating brain function and influencing mood and cognition through complex molecular mechanisms, although the strength of supporting evidence varies considerably across individual pathways, with GLP‐1‐based mechanisms having the strongest clinical validation to date [121]. Bile acids play a central role by regulating gut microbiota and metabolic pathways, impacting low‐grade inflammation, IR, and lipid metabolism to drive MetS and related disorders [22, 122]. They also activate bile acid receptors TGR5 and FXR, which promote CD8+ T‐cell activation while modulating the release of neurotransmitters such as GABA and serotonin. TGR5 activation reduces anxiety‐like behaviors via the cAMP/PKA/CREB signaling pathway [21, 123].

Beyond neurotransmitter modulation, bile acids regulate gut hormone secretion via lipid signaling, particularly enhancing GLP‐1 activity [124]. GLP‐1, secreted by intestinal L cells, stimulates insulin secretion and suppresses glucagon release, improving glycemic control [125]. The 5‐HT/GLP‐1 circuit modulates MetS‐MD comorbidity by regulating 5‐HT neuronal excitability and GLP‐1R expression in the dorsal raphe nucleus. Serotonin interacts with hypothalamic proopiomelanocortin (POMC) neurons via 5‐HT2C receptors to regulate appetite and energy metabolism alongside GLP‐1 [126]. Conversely, GLP‐1 activates GLP‐1R in the brainstem and hypothalamus to influence 5‐HT circuits, enhancing insulin secretion and appetite regulation [127]. Semaglutide restores the 5‐HT/GLP‐1 circuit balance, improving social behavior and metabolic profiles [128]. Within the gut–brain axis, the pathways described do not carry equivalent evidential weight and are better presented as a hierarchy. At the mechanistic tier, characterized primarily in rodent models and intestinal cell lines, TGR5 activation suppresses neuroinflammation and depressive‐like behavior via the cAMP/PKA/CREB cascade, while FXR shapes anxiety‐related behaviors through modulation of GABAergic and other neurotransmitter systems in knockout mouse studies [22, 129]. At an intermediate tier, paracrine crosstalk between intestinal enterochromaffin cells and GLP‐1‐secreting L‐cell converges on vagal afferent pathways, a serotonin‐GLP‐1 circuit supported by rodent experiments and intestinal organoid models but not yet evaluated as an independent mood intervention in prospective trials [130]. The most translationally mature evidence concerns systemic GLP‐1 receptor agonism: agents including liraglutide and semaglutide produced modest but statistically significant reductions in depression rating scores in a meta‐analysis of randomized controlled trials in adults with T2D, establishing GLP‐1 signaling as the only node in this axis with replicated clinical evidence for affective benefit [131].

Beyond the bile acid–GLP‐1 signaling cascade, TMAO has emerged as a second gut‐derived metabolite that mechanistically bridges cardiometabolic disease and mood dysregulation. TMAO is produced when gut bacteria catabolize dietary choline, L‐carnitine, and phosphatidylcholine to TMA, which is subsequently oxidized to TMAO by hepatic flavin‐containing monooxygenase 3 (FMO3). Landmark prospective clinical studies demonstrated that elevated plasma TMAO independently predicts major adverse cardiovascular events and all‐cause mortality, establishing it as a robust biomarker of cardiovascular risk [132, 133]. Subsequent meta‐analyses confirmed that elevated circulating TMAO associates with increased risks of heart failure and atherosclerosis, with proatherogenic mechanisms including NLRP3 inflammasome activation, enhanced platelet hyperreactivity, and endothelial dysfunction [134]. In the context of HTA, TMAO crosses the blood–brain barrier and induces central neuroinflammation and oxidative stress; inhibition of TMAO synthesis in rats fed a high‐salt diet attenuated sympathetic excitation and reduced blood pressure by suppressing neuroinflammation, identifying a gut–brain–cardiovascular axis through which dietary patterns modulate both vascular and central nervous system tone [135].

**Figure 2:**
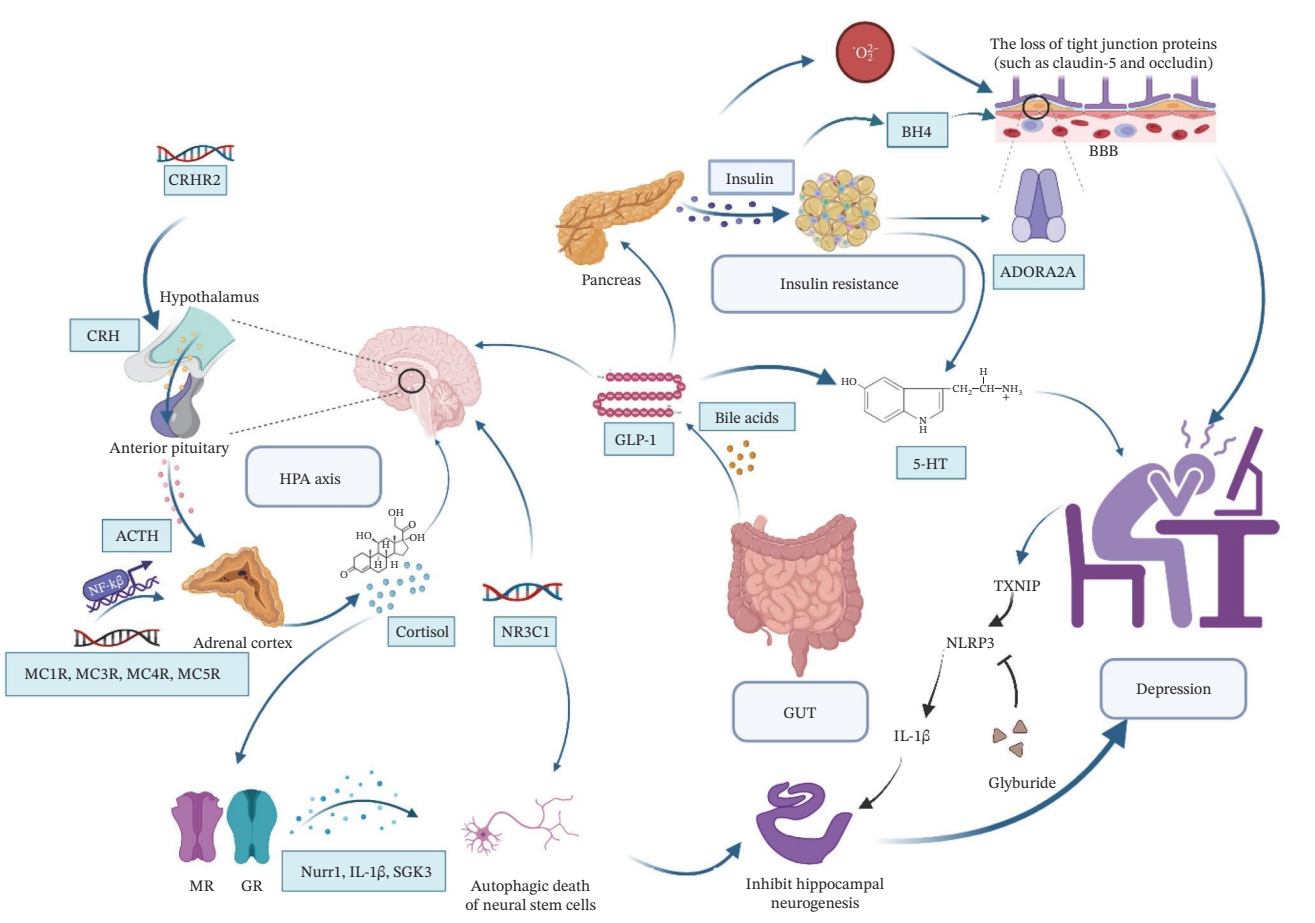
Schematic diagram of the mechanisms underlying comorbid depression in T2D. 5‐HT, 5‐hydroxytryptamine; ACTH, adrenocorticotropic hormone; BBB, blood–brain barrier; BH4, tetrahydrobiopterin; CRH, corticotropin‐releasing hormone; GLP‐1, glucagon‐like peptide‐1; GR, glucocorticoid receptor; HPA, hypothalamic–pituitary–adrenal axis; MR, mineralocorticoid receptor.

In a post‐stroke depression model, TMAO dose‐dependently aggravated depression‐like behaviors via ROS‐p38/MAPK signaling, linking cardiovascular injury, gut dysbiosis, and subsequent mood deterioration within a unified biological cascade [136]. In preclinical models, elevated TMAO induced hippocampal neuroinflammation characterized by increased microglial activation, upregulated TNF‐α and IL‐1β, and TLR‐4/NF‐κB/MyD88 pathway activation, effects mechanistically convergent with the NLRP3‐mediated neuroinflammatory pathways implicated in diabetes‐depression comorbidity described above [137]. Taken together, TMAO constitutes a gut‐derived molecular node that converges cardiometabolic pathology and mood dysregulation: it is elevated by the same dietary patterns and gut dysbiosis states that characterize MetS, HTA, and heart failure while simultaneously promoting central neuroinflammation and depressive vulnerability. The evidence linking TMAO directly to depression in humans, however, remains largely cross‐sectional and associative; the specific neuroinflammatory mechanisms are defined primarily in rodent models, and interventional trials targeting TMAO synthesis in patients with comorbid MD and CVD are lacking. The mechanisms of diabetes‐depression comorbidity are illustrated in Figure 2.

### 3.4. Gut Microbiome

The comorbidity of obesity and MD is closely linked to multiple genetic variants, supported by convergent evidence from candidate‐gene studies and GWAS, particularly in fat‐mass and obesity‐associated genes, MC4R, serotonin transporter, and catechol‐O‐methyltransferase genes [138–141]. Additionally, epigenetic modifications such as DNA methylation play significant roles in their susceptibility and pathogenesis [142, 143]. Chronic stress activates hypothalamic paraventricular nucleus neurons, increasing the sympathetic innervation of white adipose tissue and altering adipokine expression, including leptin, adiponectin, Angptl4, Sfrp5, and Fbn1, through β‐adrenergic receptor signaling. These changes promote depression‐like behaviors and exacerbate IR [144]. In obese mice, visceral adipose tissue (VAT)‐derived extracellular vesicles suppress the CREB‐BDNF signaling pathway through miR‐140‐5p, inducing synaptic damage in the hippocampus and increasing stress susceptibility, thereby driving depression‐like behaviors [145]. Epidemiological associations between obesity and depression are robustly established in large human cohorts, and shared genetic variants provide further human‐level evidence for a common predisposition. The mechanistic pathways detailed here, including adipose‐derived extracellular vesicles carrying miR‐140‐5p, stress‐induced β‐adrenergic reprogramming of white adipose tissue, and hepatic vagal neuron involvement, are characterized in rodent models and represent plausible but as‐yet‐unvalidated mechanisms in humans. Liver‐innervating vagal sensory neurons are critical in obesity‐anxiety comorbidity. Animals lacking these neurons exhibit increased energy expenditure, reduced weight and hepatic lipid accumulation, and markedly diminished anxiety‐like behaviors [146].

Obesity development is strongly associated with gut microbiome dysbiosis [147]. Emotional overeating is considered a primary driver of MD‐induced obesity, perpetuating the vicious cycle between obesity and MD [148]. In rodent models, high‐fat diets disrupt gut microbiota, activate lipopolysaccharide (LPS), and trigger proinflammatory cytokine release (IL‐6, TNF‐α, and IL‐1β), leading to neuroinflammation and promoting cooccurrence of depression and obesity [149]. IL‐6 enhances GLP‐1 secretion, activates the JAK2‐STAT3 pathway to regulate glycemic homeostasis, and modulates appetite and energy metabolism via hypothalamic and brainstem circuits, thereby attenuating obesity progression [150]. JAK2‐STAT3 activation also regulates neural stem cell differentiation, promoting neurogenesis and cognitive improvement [151]. Gut microbiota‐derived short‐chain fatty acids—acetate, propionate, and butyrate—preserve intestinal barrier integrity by activating GPR41/GPR43 receptors, mitigating systemic inflammation, modulating tryptophan metabolism and neurotransmitter levels, and regulating moods [152, 153]. Butyrate specifically enhances granzyme B expression in Th1 cells via HDAC inhibition and GPR43 activation, fostering immune tolerance and reducing intestinal inflammation, which subsequently modulates central nervous system immune responses [154]. Butyrate also binds PPARγ to activate its transcriptional activity, increasing ST2+ Treg cell populations in VAT and ameliorating VAT inflammation [155]. Gut dysbiosis further impairs immune cell functions, including CD39+ quiescent Tregs and IgD+ CD38 naïve B cells, while dysregulating inflammatory mediators such as MCSF, GDNF, and CD40, thereby mediating anxiety and MDD pathogenesis [156, 157]. Human observational data confirm gut microbiome dysbiosis in both obesity and depression, and Mendelian randomization analyses provide preliminary causal evidence linking gut immune mediators to anxiety disorders [156, 157]. The specific molecular circuits described in this section, including LPS‐triggered neuroinflammation under high‐fat conditions, SCFA/GPR receptor modulation of mood, and butyrate‐mediated HDAC inhibition regulating adipose Treg populations, are established in rodent and cell culture models and remain preclinical. Clinical trials examining these circuits as intervention targets in comorbid populations are lacking.

Chronic stress exacerbates vitamin B6 metabolism disturbances via gut dysbiosis, characterized by reduced 4‐pyridoxic acid and 4‐pyridoxate levels [158]. Beyond the obesity‐focused mechanisms described above, gut dysbiosis constitutes an equally critical bridge linking CVD and MD, extending the gut–brain axis into a broader gut–brain–heart axis. Profiling of gut microbial composition reveals a shared dysbiotic signature across CAD and anxiety/depression: jointly upregulated pathobionts, including *Staphylococcus*, *Escherichia coli*, *Helicobacter pylori*, and Shigella, alongside jointly depleted beneficial taxa, such as Prevotella, Lactobacillus, *Faecalibacterium prausnitzii*, Collinsella, and Bifidobacterium, are consistently identified in both conditions, implicating a common microbial substrate for their comorbidity [159]. *F. prausnitzii*, reduced in both CVD and depression, is a principal butyrate producer whose deficiency withdraws anti‐inflammatory SCFA signaling simultaneously from the gut epithelium and the central nervous system, mechanistically connecting endothelial inflammation and neuroinflammation within a single microbial deficit [160]. Depression‐induced gut dysbiosis further disrupts tryptophan metabolism, depleting the cardioprotective and neuroprotective metabolite, IPA. In a chronic social defeat stress model, depression‐associated reduction in IPA exacerbated myocardial ischemia/reperfusion injury by permitting ferroptosis in cardiomyocytes through impairment of the NRF2/System xc^-^/GPX4 axis; fecal microbiota transplantation from stressed donors replicated this cardiac vulnerability, and IPA supplementation reversed it [160]. This brain–gut–heart axis positions depression‐driven microbial depletion of IPA as a direct mechanistic route through which MDs worsen cardiac outcomes, complementing the TMAO‐mediated neuroinflammatory pathway described above and together framing tryptophan catabolism as a transdiagnostic metabolite axis. A third distinct gut‐derived metabolite, phenylacetylglutamine (PAGln), synthesized from dietary phenylalanine by intestinal bacteria and conjugated hepatically, further bridges CVD and neuroendocrine dysregulation. Prospective clinical cohort data demonstrate that elevated plasma PAGln independently predicts major adverse cardiovascular events, heart failure, and coronary heart disease [161]; mechanistically, PAGln acts as a negative allosteric modulator of β_2_‐adrenergic receptors (β_2_ARs) and activates platelet α_2_A/α_2_B‐adrenergic receptors to enhance prothrombotic reactivity [162]. Because β_2_‐adrenergic signaling is also a key node in stress‐mediated sympathoadrenal activation and mood regulation, PAGln‐driven attenuation of β_2_AR responsiveness in cardiomyocytes is mechanistically convergent with the β‐adrenergic reprogramming of white adipose tissue described under chronic stress, and this metabolite may simultaneously impair cardiac contractile reserve and amplify the sympathoneuroendocrine dysregulation implicated in MD. These findings collectively establish a hierarchy of gut‐derived metabolites, with IPA protective and depleted by depression, TMAO harmful and elevated by dysbiosis, and PAGln harmful and amplifying adrenergic cardiovascular risk, that operate in concert across the gut–brain–heart axis to sustain the bidirectional comorbidity of CVD and MD. The shared microbial taxonomic alterations and metabolomic signatures are documented in human‐observational studies; the specific ferroptosis, β_2_AR‐allosteric, and NRF2‐mediated mechanistic pathways remain primarily established in preclinical models and require translational validation.

**Figure 3:**
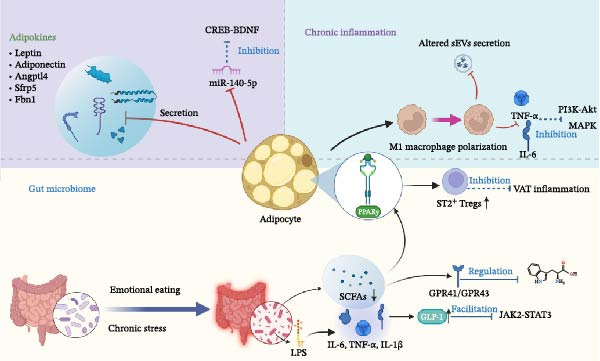
Schematic diagram of adipocyte‐centered crosstalk linking chronic stress, gut dysbiosis, and metabolic‐inflammation axis.

Vitamin B6 supplementation restores absorption by upregulating intestinal epithelial alkaline phosphatase, alleviating stress‐induced behavioral abnormalities and inflammation [163]. The relevant mechanisms are illustrated in Figure 3.

### 3.5. Chronic Inflammation

Convergent human and animal data demonstrate that obesity triggers chronic inflammation in the adipose tissue [164], marked by M1 macrophage polarization and altered secretion of small extracellular vesicles. Macrophage‐derived proinflammatory cytokines disrupt insulin signaling, exacerbating IR and glycemic dysregulation [165–167]. Obesity‐induced inflammation modulates transient receptor potential V1 signaling, reducing its expression in the hippocampus and medial prefrontal cortex while increasing it in the hypothalamus and amygdala. These changes elevate TNF‐α and IL‐6 levels, impair PI3K‐Akt and MAPK neurotrophic pathways, and ultimately drive depressive symptoms [168–170]. Inflammation also activates microglia, amplifying proinflammatory cytokine release and disrupting neural circuits—particularly connections between the anterior cingulate cortex and nucleus accumbens—thereby perturbing mood and reward processing [171].

The central nervous system and periphery interact dynamically via boundary immune regions (meninges, choroid plexus, and skull bone marrow), coordinating cerebrospinal fluid circulation, lymphatic drainage, and immune cell trafficking to influence neuronal repair, synaptic pruning, and immune surveillance [172]. Peripheral inflammation alters cerebral neurotransmitter metabolism, suppressing serotonin synthesis by diverting tryptophan toward kynurenine via indoleamine‐2,3‐dioxygenase. This generates neurotoxic quinolinic acid, worsening depressive symptoms [23]. Neuronal GHSR deficiency suppresses neuroinflammation by downregulating MAPK‐autophagy signaling [173]. NAR alleviates obesity‐driven adipose inflammation and improves insulin sensitivity via selective LXRβ activation [174]. Elevated ANGPTL8 in obesity exacerbates comorbidity [175]; inhibiting ANGPLT8 through its receptor PirB reduces hippocampal leptin and proinflammatory cytokine expression while restoring synaptic/axonal markers (Gap‐43 and Snap‐25), thereby ameliorating comorbidity [19, 20, 176, 177]. Elevated circulating proinflammatory cytokines (IL‐6, TNF‐α, and IL‐1β) and adipose macrophage polarization in humans with comorbid obesity and depression provide clinical grounding for an inflammatory hypothesis. Elevated serum ANGPTL8 has also been documented in human obesity. However, the neurospecific mechanisms described above, including TRPV1 modulation in limbic and hypothalamic circuits, microglial disruption of cortico‐accumbal connectivity, GHSR‐MAPK regulation of neuroinflammation, and PirB‐mediated hippocampal synaptic rescue, are defined exclusively in animal models. Their causal relevance to depressive symptoms in humans has not been directly demonstrated.

Beyond the obesity‐centric inflammatory pathways described above, the NLRP3 inflammasome constitutes a transdiagnostic inflammatory node that mechanistically bridges CVD and MD across the full cardiometabolic spectrum. In CVD, NLRP3 activation by cholesterol crystals, reactive oxygen species, and damage‐associated molecular patterns (DAMPs) drives caspase‐1‐mediated cleavage of pro‐IL‐1β and pro‐IL‐18, precipitating pyroptotic cardiomyocyte death, atherosclerotic plaque instability, and adverse ventricular remodeling [178]. Clinically, the CANTOS trial provided landmark proof‐of‐concept that IL‐1β inhibition with canakinumab significantly reduced recurrent major adverse cardiovascular events in post‐myocardial infarction patients independently of lipid lowering, directly validating the inflammatory hypothesis of atherothrombosis in humans [179]. Paralleling these cardiovascular effects, NLRP3 is persistently activated in patients with MDD, where microglial NLRP3‐caspase‐1 signaling drives the release of IL‐1β and IL‐18, induces hippocampal neuronal pyroptosis, disrupts neurotrophic‐factor support, and sustains the HPA‐axis hyperreactivity that reinforces depressive pathology; in murine models, NLRP3 deletion abolishes chronic stress‐induced microglial activation and depressive‐like behavior [180]. This shared inflammasome activation is further amplified by the convergence of metabolic stressors: in HTA, NLRP3‐driven IL‐1β production in vascular smooth muscle cells promotes phenotypic transformation and arterial remodeling, while in obesity‐driven MetS, adipose NLRP3 activation impairs insulin signaling and accelerates endothelial dysfunction, linking cardiometabolic and neuropsychiatric diseases within a unified inflammatory cascade [178]. A second shared inflammatory pathway is IDO‐mediated tryptophan catabolism via the KP. In atherosclerosis, IDO1 is upregulated in inflamed arterial plaques, and elevated kynurenine/tryptophan ratios positively correlate with carotid intima‐media thickness and adverse prognosis in coronary artery disease [181]. In MD, the same IDO‐driven diversion of tryptophan away from serotonin synthesis toward neurotoxic quinolinic acid underpins neuroinflammatory depressive pathology, as described under “Chronic Inflammation” and detailed in other sections of this review. Crucially, the kynurenine metabolite profile differs in its cardiovascular vs. neuropsychiatric consequences: kynurenic acid and certain KP intermediates may exert vasodilatory and plaque‐stabilizing effects, while quinolinic acid preferentially drives neurotoxicity, suggesting that the KP represents not simply a shared harmful pathway but a branched metabolite axis whose organ‐specific outputs depend on the local enzymatic context. This distinction is clinically important: it implies that nonselective IDO inhibition, while conceptually appealing for both CVD and MD, may carry offsetting risks requiring disease‐context‐specific targeting. The NLRP3‐IL‐1β pathway is the most translationally advanced of these shared inflammatory nodes, with proof‐of‐concept human data from CANTOS; IDO/KP alterations in both CVD and MD are documented in human cohort and biomarker studies, but interventional evidence in comorbid populations remains absent [182].

## 4. Disease‐Specific Mechanisms Linking MD to Individual Cardiometabolic Conditions

### 4.1. CVD and MD

CVD and MD share overlapping pathological mechanisms. HTA, coronary heart disease, heart failure, and atrial fibrillation induce structural changes in the cerebral cortex, primarily affecting emotion‐ and cognition‐related regions such as the frontal lobe, temporal lobe, and cingulate gyrus [183]. Mendelian randomization studies on heart failure and cortical structure support these findings, demonstrating that heart failure significantly reduces cortical thickness and surface area in the caudal middle frontal, insular, precuneus, and superior parietal regions—changes strongly linked to cognitive dysfunction and MD [184]. CVD promotes chronic cerebral hypoperfusion, driving vascular remodeling and endothelial dysfunction [185], which alter the cortical surface area and thickness, thereby elevating MD risk. Conversely, abnormal cortical structures can predict CVD onset, further evidencing bidirectional heart–brain and brain–heart axis interactions [186].

#### 4.1.1. BDNF Levels

Lower serum BDNF levels correlate with a higher risk of depression in patients with CVD [187]. Reduced BDNF serves as a biomarker for depressive symptoms in CVD [188–190], with low BDNF levels implicated in MD pathogenesis [191, 192]. BDNF primarily mediates anxiety‐ and depression‐like behaviors via the BDNF/TrkB pathway. Dysfunction of the suprachiasmatic nucleus activates BDNF–TrkB signaling, modulating striatal neuronal activity to drive anxiety and depression [193, 194]. Chronic stress downregulates hippocampal SorCS2 expression, impairing its binding to TrkB and suppressing the ERK/AKT/CREB pathway, thereby inducing depression‐like behaviors [195]. Deficits in BDNF/TrkB signaling contribute to both cerebral and cardiac disorders. BDNF/TrkB activation of downstream ERK/AKT pathways enhances cardiomyocyte proliferation, reduces apoptosis, and mitigates ischemic‐reperfusion injury in ischemic heart disease [196, 197]. The Sigma‐1 receptor promotes BDNF synthesis by activating CREB transcription factors [198], regulates endoplasmic reticulum‐mitochondrial crosstalk to maintain neuronal homeostasis, synergizes with NMDAR signaling to enhance synaptic plasticity [199], and suppresses neuroinflammation to protect BDNF‐mediated neural repair [200]. Additionally, Sigma‐1 receptors inhibit astrocytic‐NF‐κB inflammatory signaling, curbing neuroinflammatory amplification and exerting rapid antidepressant effects [201]. Human prospective and cross‐sectional studies consistently find that lower serum BDNF levels are linked to more depressive symptoms in CVD patients, making reduced BDNF the most supported biomarker in this context. Rodent and in vitro work has identified several downstream mechanisms, such as SorCS2–TrkB interaction under chronic stress, Sigma‐1 receptor regulation of ER–mitochondrial crosstalk, and astrocytic‐NF‐κB suppression. While biologically plausible, these mechanisms should still be seen as hypotheses awaiting human validation.

#### 4.1.2. Renin–Angiotensin–Aldosterone System (RAAS)

**Figure 4:**
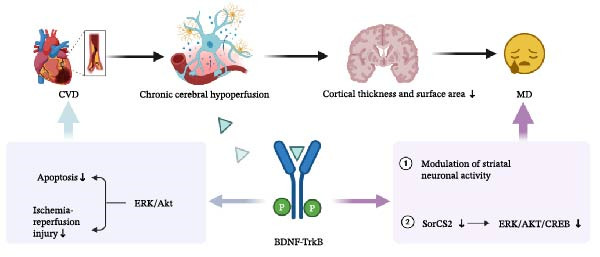
Schematic diagram of RAAS‐mediated mechanisms in HTN and depression comorbidity. CVD, cardiovascular disease; HTN, hypertension; MD, mood disorder; RAAS, renin–angiotensin–aldosterone system.

The coexistence of HTA and MD is closely tied to RAAS dysregulation [202, 203]. Aldosterone plays a pivotal role in HTA development and maintenance [204]. Individuals with comorbid depression and HTA exhibit significantly higher aldosterone levels compared to those with only HTA or healthy controls [205, 206]. Aldosterone acts via MRs in brain regions critical for emotion, memory, and decision‐making, including the hippocampus, amygdala, and prefrontal cortex [207]. Angiotensin II (Ang II) activates AT1A receptors in the brain, stimulating CRH release and HPA‐axis activation while upregulating β‐adrenergic receptor production, thereby modulating stress responses and anxiety. Ang II also enhances NADPH oxidase activity, triggering oxidative stress and activating MAPK/NF‐κB pathways [208], which drive proinflammatory cytokine release, neuronal damage, and behavioral alterations [209]. Aldosterone exacerbates oxidative stress and neuroinflammation by activating spinal ACE/Ang II/AT1R signaling, inducing NOX2‐mediated oxidative stress [210]. Elevated aldosterone correlates with oxidative markers like 8‐isoprostane, further explaining its role in depressive and anxiety symptoms [211]. KCNJ5 mutations, associated with primary aldosteronism, promote 18‐oxocortisol and 18‐hydroxycortisol secretion, which influence mood via MR stimulation [212, 213]. The role of the RAAS system in HTA and MD comorbidity is shown in Figure 4. The clinical association between aldosterone excess and comorbid depression is substantiated by human case–control and population‐based studies, and the mood benefits of adrenalectomy or MR antagonists in primary aldosteronism are supported by clinical evidence [214]. The molecular mechanisms linking Ang II to neuroinflammation via NADPH oxidase, MAPK/NF‐κB, and NOX2‐driven oxidative stress are established in animal models and in vitro systems; their direct contribution to depressive symptoms in hypertensive humans has not been demonstrated in interventional studies.

Gender differences also shape aldosterone’s role in MD. Women with elevated aldosterone exhibit more pronounced mood fluctuations and anxiety, suggesting heightened susceptibility to its adverse effects compared to those of men [215].

### 4.2. Dyslipidemia and MD

Low HDL‐C levels show a robust association with depression, particularly regarding symptom duration and severity [216–218]. HDL‐C and depression exhibit a linear inverse relationship, with lower HDL‐C conferring a higher depression risk in women and obese populations [219]. HL (HDL, non‐HDL cholesterol, and triglycerides) significantly correlates with effort–reward task performance in patients with MDD. Higher HDL and triglyceride levels associate with increased effort discounting, independent of glycemia and IR [220]. MDD patients display significantly lower total cholesterol and elevated sodium voltage‐gated channel α subunit 11A (SCN11A) concentrations. The MANF/EWSR1/ANXA6 pathway may bridge hypolipidemia and MDD, serving as a potential diagnostic biomarker [221–223]. The inverse association between HDL‐C and depression is among the more consistent lipid–mood findings across independent human cohorts. The MANF/EWSR1/ANXA6 pathway as a molecular bridge between hypolipidemia and MDD is an emerging hypothesis derived from transcriptomic profiling of patient samples; it requires replication and experimental validation before it can be considered an established mechanism or a clinically actionable biomarker.

## 5. Sex‐Specific Mechanisms in MD–CD Comorbidity

Sex is one of the most consistent biological modifiers of MD–CD comorbidity, acting systematically across the neuroendocrine, metabolic, and cardiovascular pathways described earlier. After puberty, women have roughly twice the lifetime prevalence of depression as that of men, implicating ovarian hormones as upstream regulators of shared vulnerability rather than mere demographic confounders. Estrogen operates at several mechanistic nodes relevant to this review: it suppresses corticotropin‐releasing hormone transcription and reduces glucocorticoid‐receptor sensitivity in limbic circuits, thus moderating HPA ‐axis reactivity; it enhances serotonergic transmission by upregulating tryptophan hydroxylase‐2 (TPH2) while inhibiting MAO‐A; and it limits neuroinflammatory amplification through suppression of microglial NF‐κB signaling and decreased proinflammatory cytokine output. Physiological transitions marked by rapid estrogen withdrawal or sharp fluctuation—including the late luteal phase, the postpartum period, and the perimenopausal transition—therefore disrupt these regulatory functions in concert, substantially lowering the threshold for depressive episodes and worsening cardiometabolic risk [224].

These hormonal influences intersect with metabolic comorbidity in a sex‐dependent way. A multiancestry, bidirectional Mendelian randomization study with sex‐stratified analysis showed that the causal pathway from depression to type 2 diabetes is significantly stronger in females than in males, partly mediated by body mass index and smoking behavior; the reverse direction—T2D causally affecting depression—was not supported in either sex [225]. Independent body‐composition analyses using dual‐energy X‐ray absorptiometry in the NHANES cohort found that fat‐mass index was more strongly tied to depressive symptoms in women, and a statistically significant multiplicative interaction between fat mass and appendicular lean mass on depression risk appeared only in females, not in males [226]. These findings align with estrogen’s role in regulating adipose tissue distribution and hypothalamic leptin‐receptor signaling: postmenopausal estrogen deficiency promotes visceral fat accumulation, hyperleptinemia, and central leptin resistance, each of which can independently suppress hippocampal neurogenesis and heighten HPA‐axis hyperactivity, thereby deepening the reciprocal link between metabolic dysfunction and depression in women.

In cardiovascular and lipid domains, sex continues to shape the key associations described above. As noted under the RAAS, women with elevated aldosterone exhibit more pronounced mood swings and anxiety than men [215], suggesting either greater MR sensitivity or a stronger neuroinflammatory response to aldosterone excess in female limbic circuits. At the lipid–mood interface, the inverse association between HDL‐C and depression is stronger in women and in individuals with obesity [219]. A meta‐analysis of 49 cross‐sectional studies involving nearly 400,000 participants found that depressed women had about 1.95‐fold higher odds of meeting MetS criteria (OR = 1.95; 95% CI: 1.38–2.74) compared with nondepressed women, whereas no statistically significant association emerged among men [227]. These patterns are consistent with estrogen’s cardioprotective contributions to reverse cholesterol transport and lipid homeostasis—effects that wane progressively during and after the menopausal transition, simultaneously raising CVD risk and depression susceptibility through converging lipid‐inflammatory pathways.

Across these domains, sex differences converge on a set of estrogen‐regulated molecular nodes: glucocorticoid‐receptor expression and nuclear translocation in limbic circuits, hypothalamic and hippocampal leptin‐receptor signaling, and microglial NF‐κB‐driven inflammatory tone. These overlapping mechanisms place estrogen as a transdiagnostic moderator of the neuroendocrine, metabolic, and cardiometabolic pathways underlying MD–CD comorbidity. It should be clearly acknowledged, however, that most sex‐stratified evidence to date comes from cross‐sectional observational studies, post‐hoc subgroup analyses, and Mendelian randomization studies not designed a priori for sex‐specific mechanistic inference. The estrogen‐centered framework presented here is biologically coherent but remains largely inferential. Dedicated prospective studies that enroll participants across hormonal transition states and include prespecified sex‐stratified mechanistic and clinical endpoints are needed before sex‐specific causal pathways can be considered established. Nevertheless, recognizing sex as a biological moderator is immediately actionable: it should inform cardiometabolic screening thresholds for women presenting with depressive disorders, guide risk stratification, and be essential for designing interventional trials with sufficient statistical power to detect sex‐specific treatment effects.

## 6. Beyond Cardiometabolic Disease: Other Chronic Somatic Comorbidities

### 6.1. OA and MD

The brain–joint axis plays a pivotal role in OA–MD comorbidity, with neuroendocrine dysregulation—including leptin signaling abnormalities, central sensitization, and neuroimmune pathway disturbances—potentially driving their bidirectional association [228, 229]. Prospective studies reveal that recurrent depression independently increases the risk of hyperleptinemia 2.9‐fold within 18 months, irrespective of weight changes [230]. Elevated leptin directly activates chondrocyte JAK/STAT and NF‐κB pathways, inducing proinflammatory cytokine release and accelerating cartilage degradation [231–233]. Adipokines such as adiponectin, resistin, and visfatin further contribute to joint destruction by regulating MMPs and inflammasome pathways [234, 235]. Beyond adipokine signaling, chronic peripheral inflammation provides a second, partly independent route linking OA to mood disturbances. Sustained release of proinflammatory cytokines (IL‐1β, IL‐6, and TNF‐α) from osteoarthritic joints increases blood–brain barrier permeability and facilitates the entry of circulating inflammatory mediators into the central nervous system, where they provoke microglial activation, oxidative stress, and synaptic dysfunction—core features of the emerging brain–joint axis framework [236]. In experimental models, induction of knee OA is itself sufficient to trigger astrocyte and microglial activation and to accelerate neurodegenerative pathology in the brain, demonstrating that localized joint inflammation can drive central neuroinflammatory change in vivo [237]. Peripheral cytokine elevation additionally activates indoleamine‐2,3‐dioxygenase, diverting tryptophan away from serotonin synthesis toward neurotoxic kynurenine metabolites; consistent with this, KP metabolites are measurably altered in the synovial fluid and serum of patients with osteoarthritis [238, 239]. A further clinically prominent feature is central sensitization: in a subset of OA patients, maladaptive neuroplastic changes in supraspinal pain‐processing regions amplify and perpetuate pain independently of the degree of structural joint damage, and this nociplastic phenotype is closely intertwined with depressive and anxiety symptoms, helping to explain why pain severity in OA often correlates poorly with radiographic findings [236].

Population‐based studies show a clear epidemiological link between osteoarthritis and depression. The controlled SPIRR‐CAD trial further supports a prospective connection from recurrent depression to hyperleptinemia, offering credible human causal evidence. At the chondrocyte level, mechanisms such as leptin‐driven JAK/STAT and NF‐κB activation and adipokine regulation of MMP pathways have been characterized mainly in cell culture models. Whether these pathways mediate depressive pathology in the joint–brain axis in humans is yet to be tested.

### 6.2. Neurological Disorders and MD

Psychosomatic comorbidity is also prevalent in neurological disorders, where shared neurodegenerative, neuroinflammatory, and neurotransmitter mechanisms frequently render depression an early and integral feature of the disease rather than a purely reactive response. Depressive symptoms in dementia with Lewy bodies are directly linked to dopaminergic dysfunction in the subgenual anterior cingulate cortex, characterized by reduced dopaminergic fiber density and downregulated DAT, TH, and DRD3 expressions. Abnormal synaptic accumulation of phosphorylated α‐synuclein disrupts synaptic function by interfering with the soluble NSF attachment protein receptor (SNARE) complex [240]. Depression is one of the most common nonmotor symptoms of Parkinson’s disease (PD), often appearing before motor signs. α‑Synuclein has been proposed as a molecular point of convergence between the two conditions. Pathological α‑synuclein accumulates in brainstem raphe nuclei during the early Braak stages, disrupting serotonergic neurotransmission. Together with the degeneration of nigrostriatal dopaminergic neurons and reduced dopamine transporter availability, this contributes to depressed mood before classic motor symptoms emerge [241]. Notably, targeted overexpression of human α‑synuclein restricted to raphe serotonergic neurons is enough to produce a depressive‑like phenotype in mice, accompanied by axonal damage, BDNF deficits, and disrupted serotonergic signaling. Suppressing α‑synuclein synthesis reverses these deficits, providing direct causal evidence that α‑synuclein pathology can drive depression through serotonergic circuit dysfunction (Miquel‐Rio et al. 2022). However, the convergent‑pathway and genomic analyses underlying this model come mainly from secondary transcriptomic data and animal studies; the causal mechanistic steps still need confirmation in human longitudinal cohorts.

Depression is also very common in multiple sclerosis (MS), with lifetime estimates approaching 50%. It is increasingly viewed as biologically embedded in the immunopathology of the disease rather than simply a psychological reaction to disability. The same neuroinflammatory processes that cause demyelination—microglial activation and elevated proinflammatory cytokines, including IL‑6, IL‑1β, and TNF‑α—also activate the HPA axis, reduce serotonergic neurotransmission, and impair neurotrophic support, mechanisms shared with idiopathic depression. A systematic review and meta‑analysis confirmed that circulating inflammatory protein levels are associated with depression severity in people with MS, lending human‑level support to this immune‑mediated model, though the directionality and treatment implications remain unclear [242].

### 6.3. Psoriasis and MDD

Psoriasis and depression are increasingly seen as linked through a bidirectional brain–skin axis, in which shared inflammatory, neuroendocrine, and immune dysregulation sustains both conditions [243]. In psoriatic skin, activated dendritic cells release IL‑23 and TNF‑α, driving Th17 and γδ T‑cell differentiation to generate IL‑17A, IL‑17F, and IL‑22, creating a self‑perpetuating cutaneous inflammatory loop. The resulting systemic low‑grade inflammation, marked by elevated circulating IL‑6, TNF‑α, and IL‑17, can compromise blood–brain barrier integrity and promote neuroinflammation, microglial activation, and disturbed neurotransmission. Peripheral cytokines further activate indoleamine‑2,3‑dioxygenase, diverting tryptophan toward kynurenine metabolites and reducing serotonin synthesis [243, 244]. Th17 cells may also act directly on brain‑resident cells, causing calcium‑mediated neuronal toxicity and inducing microglial indoleamine‑2,3‑dioxygenase. These peripheral signals intersect with HPA‑axis dysfunction and, via the gut–brain–skin axis, with microbiota dysbiosis. Reduced short‑chain fatty acid production and increased LPS translocation amplify systemic and neuroinflammatory tone, reinforcing the comorbidity [244]. At a more specific molecular level, the comorbidity mechanism between psoriasis and MDD involves CD19‑mediated B‐cell immune dysregulation, which modulates inflammation through the PPARγ/β‑catenin/Wnt3a pathway. CD19 upregulation promotes immune cell activation, while PPARγ inhibition attenuates inflammation, and targeting CD19 ameliorates this comorbidity [245]. The relevant mechanisms are summarized in Table 2. Whereas the systemic inflammatory and brain–skin‐axis framework is supported by convergent human cohort, neuroimaging, and biomarker data, the CD19/PPARγ pathway is identified in a single translational study and remains preclinical. This pathway is identified in a single translational study and remains preclinical; independent replication and clinical validation are required before CD19 can be proposed as a therapeutic target for psoriasis–MDD comorbidity.

**Table 2:** Mechanisms underlying comorbidity between mood disorders and chronic somatic diseases.

Shared mechanism	Principal mediators/effectors	Conditions implicated	Strength and nature of evidence	Representative references
Shared mechanisms underlying comorbidity between mood disorders and chronic somatic diseases				
HPA axis/glucocorticoid dysregulation	↑ACTH, ↑cortisol; gluco‐ and mineralocorticoid receptors NR3C1, CRHR2, FKBP5, MC1R–MC5R; 11β‐HSD1 Nurr1, SGK3, IL‐1β, NF‐κB	T2D, MetS, obesity, hypertension, and CVD	Strong, replicated: ↑ACTH/cortisol with impaired feedback in T2D and ↑cortisol output in MetS across independent cohorts MR supports cortisol→hypertension and depression→T2D; the cortisol→depression direction is explicitly null Candidate genes (NR3C1, CRHR2, MC‐R, and FKBP5) from small/single cohorts—need ancestry‐diverse replication	[12, 24, 77, 84–89, 95, 246]
Insulin resistance/central insulin signaling	Insulin receptor–PI3K/Akt–eNOS; MAO‐A/B Adora2a; claudin‐5, occludin; BH4/Nox2; Nrf2/HDAC3 Leptin–JAK2/STAT3; FGF21; endothelin‐1	T2D, obesity, hypertension, and CVD	Strong: peripheral IR (fasting insulin, HOMA‐IR) prospectively predicts/correlates with MDD independent of BMI (cohorts + meta‐analysis) MR, IR→hypertension and venous thromboembolism Pathway‐selective endothelial IR and leptin‐resistance neurotrophic effects mainly rodent/in vitro—need translational confirmation	[15, 17, 100, 108, 115, 118–120]
Chronic systemic inflammation/NLRP3 inflammasome	NLRP3–caspase‐1–IL‐1β/IL‐18; IL‐6, TNF‐α Cholesterol crystals, ROS, DAMPs; M1 macrophages, microglia ANGPTL8/PirB; TRPV1	Obesity, MetS, hypertension, CVD, and MDD	Human proof‐of‐concept on the CVD side (CANTOS: canakinumab/IL‐1β blockade ↓ recurrent MACE independent of lipids) ↑Circulating IL‐6/TNF‐α/IL‐1β and serum ANGPTL8 are documented in human obesity/depression Neurospecific steps (limbic TRPV1, microglial pyroptosis, and PirB hippocampal rescue) rodent only—causal human link in depression unproven	[20, 149, 163, 169, 179, 247]
IDO–kynurenine pathway	IDO1; tryptophan→kynurenine Quinolinic acid (neurotoxic) vs. kynurenic acid (vasoactive/plaque‐stabilizing) ↓Serotonin	CVD/atherosclerosis, OA, psoriasis, IBD, cancer, and MDD	Human [239] biomarker/cohort data: ↑Kyn/Trp ratio tracks carotid intima‐media thickness, CAD prognosis, and tumor‐associated depression Branched, organ‐specific outputs caution against nonselective IDO inhibition No interventional evidence in comorbid populations	[23, 181, 228, 238]
Gut microbiota and microbial metabolites	Bile acids–TGR5/FXR; GLP‐1; SCFAs (butyrate)–GPR41/43; LPS TMAO–FMO3 (harmful) and indole‐3‐propionic acid (protective) Phenylacetylglutamine–β_2_AR (harmful); *F. prausnitzii*	T2D, obesity, CVD/HF/atherosclerosis, IBD, and MDD	Most clinically mature node: GLP‐1 receptor agonists give modest but significant ↓depression scores in T2D (RCT meta‐analysis) TMAO and PAGln independently predict major adverse cardiovascular events (prospective cohorts) Taxonomic/metabolomic shifts human‐observational; ferroptosis, β_2_AR‐allosteric and NRF2 mechanisms preclinical	[22, 131–133, 160, 162]
BDNF/neurotrophic‐factor deficit	BDNF–TrkB; ERK/AKT/CREB SorCS2; Sigma‐1 receptor NF‐κB	CVD (and broadly across MD)	Reduced serum BDNF is a consistent human biomarker of depression in CVD (prospective + cross‐sectional). Downstream SorCS2–TrkB, Sigma‐1/ER–mitochondrial and astrocytic‐NF‐κB pathways rodent/in vitro—mechanistic hypotheses awaiting human validation	[187, 188, 190, 195, 201]
Disease‐specific/disease‐predominant mechanisms				
Hypertension/CVD	RAAS–aldosterone–mineralocorticoid‐receptor signaling	Aldosterone–MR; Ang II–AT1R NADPH oxidase/NOX2; MAPK/NF‐κB; 8‐isoprostane KCNJ5; 18‐oxo‐/18‐OH‐cortisol	Human case–control/population data link aldosterone excess to comorbid depression/anxiety; mood improves after adrenalectomy and (for anxiety) MR antagonism in primary aldosteronism Ang II→neuroinflammation molecular steps in rodent/in vitro. Effect more pronounced in women	[205, 206, 210, 214, 215]
Dyslipidemia	MANF/EWSR1/ANXA6 lipid–mood bridge	↓HDL‐C, ↓total cholesterol MANF, EWSR1, ANXA6; SCN11A	Inverse HDL‐C, depression association consistent across independent human cohorts (stronger in women/obesity) MANF/EWSR1/ANXA6 bridge an emerging transcriptomic hypothesis from patient samples—requires replication and experimental validation before biomarker use	[216, 217, 221, 222]
Osteoarthritis	Leptin/adipokine–chondrocyte signaling (brain–joint axis) + central sensitization	Leptin–JAK/STAT–NF‐κB; adiponectin, resistin, and visfatin MMPs; IL‐1β/IL‐6/TNF‐α; kynurenine Nociplastic (central) pain	Robust epidemiological OA, depression link; prospective SPIRR‐CAD trial shows recurrent depression→hyperleptinaemia (2.9× at 18 monts, weight‐independent) Chondrocyte JAK/STAT–MMP steps in vitro; whether they mediate mood pathology is untested in humans	[230–232, 234, 236, 237]
Parkinson’s disease/dementia with Lewy bodies	α‐Synuclein–monoaminergic (dopaminergic + serotonergic) dysfunction	α‐synuclein–SNARE complex DAT, TH, and DRD3 (dopaminergic) Raphe 5‐HT neurons; BDNF	Direct causal rodent evidence: raphe‐restricted α‐synuclein overexpression reproduces a reversible depressive‐like phenotype Human convergence is largely transcriptomic/observational; causal steps await longitudinal human cohorts	[240, 241, 248]
Psoriasis	CD19–PPARγ/β‐catenin/Wnt3a B‐cell pathway (brain–skin axis)	CD19; PPARγ/β‐catenin/Wnt3a IL‐23/IL‐17/IL‐22; TNF‐α; Th17 IDO	Systemic‐inflammation/brain–skin‐axis framework supported by convergent human cohort, neuroimaging, and biomarker data The specific CD19/PPARγ pathway rests on a single translational study—preclinical; needs independent replication before CD19 is proposed as a target	[243–245]

Abbreviations: ACTH, adrenocorticotropic hormone; Adora2a, adenosine receptor 2a; Ang II, angiotensin II; ANGPTL8, angiopoietin‐like protein 8; AT1R, angiotensin II type 1 receptor; β_2_AR, β_2_‐adrenergic receptor; BDNF, brain‐derived neurotrophic factor; BH4, tetrahydrobiopterin; CAD, coronary artery disease; CREB, cAMP‐response‐element‐binding protein; CRHR2, corticotropin‐releasing hormone receptor 2; CVD, cardiovascular disease; DAMPs, damage‐associated molecular patterns; DAT, dopamine transporter; DRD3, dopamine receptor D3; eNOS, endothelial nitric oxide synthase; ERK/AKT, extracellular‐signal‐regulated kinase/protein kinase B; *F. prausnitzii*, *Faecalibacterium prausnitzii*; FKBP5, FK506‐binding protein 5; FMO3, flavin‐containing monooxygenase 3; FXR, farnesoid X receptor; GLP‐1, glucagon‐like peptide‐1; GPR41/43, G‐protein‐coupled receptors 41/43; HDAC3, histone deacetylase 3; HDL‐C, high‐density‐lipoprotein cholesterol; HF, heart failure; HOMA‐IR, homeostatic model assessment of insulin resistance; HPA, hypothalamic–pituitary–adrenal; 11β‐HSD1, 11β‐hydroxysteroid dehydrogenase type 1; IBD, inflammatory bowel disease; IDO(1), indoleamine‐2,3‐dioxygenase (1); IL, interleukin; IR, insulin resistance; JAK/STAT, Janus kinase/signal transducer and activator of transcription; KCNJ5, potassium inwardly‐rectifying channel subfamily J member 5; LPS, lipopolysaccharide; MACE, major adverse cardiovascular events; MAO‐A/B, monoamine oxidase A/B; MAPK, mitogen‐activated protein kinase; MC1R–MC5R, melanocortin receptors 1–5; MD, mood disorders; MDD, major depressive disorder; MetS, metabolic syndrome; MMPs, matrix metalloproteinases; MR (genetics), Mendelian randomization; MR (receptor), mineralocorticoid receptor; NF‐κB, nuclear factor kappa B; NLRP3, NLR family pyrin domain‐containing 3; NOX2/Nox2, NADPH oxidase 2; NR3C1, glucocorticoid‐receptor gene; Nrf2, nuclear factor erythroid 2‐related factor 2; Nurr1, nuclear receptor related 1 protein; OA, osteoarthritis; PAGln, phenylacetylglutamine; PI3K, phosphoinositide 3‐kinase; PirB, paired immunoglobulin‐like receptor B; PPARγ, peroxisome proliferator‐activated receptor gamma; RAAS, renin–angiotensin–aldosterone system; ROS, reactive oxygen species; SCFAs, short‐chain fatty acids; SCN11A, sodium voltage‐gated channel alpha subunit 11A; SGK3, serum/glucocorticoid‐regulated kinase 3; SNARE, soluble NSF attachment protein receptor; T2D, type 2 diabetes; TGR5, Takeda G‐protein‐coupled receptor 5; TH, tyrosine hydroxylase; Th17, T‐helper‐17 cell; TMAO, trimethylamine N‐oxide; TNF‐α, tumor necrosis factor alpha; TrkB, tropomyosin receptor kinase B; TRPV1, transient receptor potential vanilloid 1; VTE, venous thromboembolism.

### 6.4. IBD and MD

IBD, encompassing Crohn’s disease and ulcerative colitis, carries a markedly elevated risk of depression and anxiety that exceeds what would be expected from the psychological burden of chronic illness alone, pointing to shared biological mechanisms centered on the microbiota–gut–brain axis [199, 249]. Chronic intestinal inflammation in IBD is driven by loss of immune tolerance to the commensal microbiota and aberrant activation of Th1 and Th17 responses, producing IFN‐γ, TNF‐α, IL‐17, and IL‐22. The accompanying gut dysbiosis reduces short‐chain fatty acid–producing taxa, compromises intestinal barrier integrity, and increases LPS translocation, generating a systemic inflammatory state that raises blood–brain barrier permeability and promotes neuroinflammation, microglial activation, and impaired hippocampal neurogenesis [250]. Because it represents a well‐defined peripheral inflammatory state, IBD has been proposed as a valuable human model for studying how chronic systemic inflammation shapes the central nervous system function and mood. While the bidirectional epidemiological association and peripheral inflammatory and microbial alterations are documented in human studies, several of the specific neuroimmune mechanisms remain characterized primarily in animal models and require clinical confirmation.

### 6.5. Cancer and MD

Depression and anxiety affect a substantial proportion of patients with cancer, and growing evidence suggests that this comorbidity is not simply a reaction to a life‑threatening diagnosis but also reflects a shared, inflammation‑centered biology [239, 251]. Tumors and the anti‑tumor immune response generate sustained systemic elevations of pro‑inflammatory cytokines, principally IL‑6, TNF‑α, and IL‑1β, which can act on the brain to produce a constellation of sickness behaviors—fatigue, anhedonia, cognitive impairment, and low mood—that overlap substantially with depressive symptoms. A central mechanistic node is cytokine‑driven induction of indoleamine‑2,3‑dioxygenase, which accelerates tryptophan catabolism along the KP, simultaneously depleting the serotonin precursor tryptophan and generating neurotoxic metabolites such as quinolinic acid. An elevated kynurenine‑to‑tryptophan ratio has accordingly been correlated with depressive symptoms and reduced quality of life in patients with solid tumors [239, 252]. The relationship appears bidirectional: depression‑associated HPA‑axis activation, sympathetic signaling, and immune dysregulation foster a pro‑inflammatory milieu that may in turn promote tumor proliferation, angiogenesis, and immune evasion through JAK‑STAT3, NF‑κB, and MAPK signaling [251]. Although the inflammatory and kynurenine‑pathway associations are supported by human biomarker studies, the evidence base remains heterogeneous and largely correlational, and rigorous interventional trials of anti‑cytokine or IDO‑targeted strategies in patients with comorbid cancer and depression are still lacking.

## 7. Therapeutic Approaches for Comorbidity

Current medical systems primarily focus on evidence‐based therapies for single diseases, with limited clinical and basic research being dedicated to comorbidity management. Existing strategies predominantly rely on disease‐specific treatments and combination therapies for comorbid conditions [253], leading to reduced therapeutic precision and healthcare resource waste [254]. The gut–brain axis has emerged as a critical pathway for comorbidity intervention, with GLP‐1 serving as a key therapeutic target [255]. The evidence base for these therapeutic approaches spans a wide spectrum of preclinical, translational, and clinical data; clearly distinguishing these tiers is essential to avoid overstating clinical readiness. In preclinical rodent models, the GLP‐1 receptor agonist exenatide activates the ERK signaling pathway, promotes M2 polarization of microglia, suppresses M1 microglial inflammatory responses [256], inhibits proinflammatory factors, and alleviates neuroinflammation and anxiety‐like behaviors by attenuating scavenger receptor A4 upregulation [257]. Whether these neuroimmune effects translate to affective benefit in humans with metabolic‐mood comorbidity has not been established in prospective clinical trials. Similarly, in rodent models of comorbid metabolic disease, the GLP‐1 analog liraglutide improves synaptic morphology in the medial prefrontal cortex and hippocampus, enhances synaptic transport‐related protein levels, and ameliorates depression‐ and anxiety‐like behaviors in these rodent models [258]; at the clinical level, a meta‐analysis of randomized controlled trials provides more directly human‐relevant evidence, demonstrating modest but statistically significant reductions in depression rating scores with GLP‐1 receptor agonists, including liraglutide, in adults with T2D [131]. In preclinical high‐fat diet mouse models, garlic‐derived exosome‐like nanoparticles train gut probiotics to release outer membrane vesicles, which cross the BBB to inhibit the cGAS‐STING pathway in microglia, reduce neuroinflammation, and activate the GLP‐1 pathway via Amuc‐1100 and P9 proteins to enhance insulin secretion, ultimately improving IR and glycemic dysregulation [259]; this approach has not been evaluated in human subjects. In a randomized placebo‐controlled pilot trial in patients with MDD, probiotics alleviate anxiety symptoms by modulating gut microbiota diversity and elevating anti‐inflammatory bacillaceae species [260]; however, this trial was small, and replication in larger comorbid populations is needed before these findings can be generalized. Tirzepatide, a dual GLP‐1/GIP receptor agonist, is clinically established to improve metabolic parameters in obesity and T2D across large randomized trials; preclinical studies additionally suggest that it mitigates obesity‐induced metabolic disorders by reducing M1 macrophage infiltration into adipose tissue and suppressing their proinflammatory cytokine secretion [261], though this specific cellular mechanism remains to be confirmed in human adipose tissue studies. Among RAAS‐targeted strategies, clinical evidence from a patient cohort demonstrates that adrenalectomy significantly improves depressive symptoms by lowering aldosterone levels, whereas MR antagonists effectively reduce anxiety but exhibit limited antidepressant efficacy [214]. In a nonrandomized single‐arm clinical trial of 41 patients with primary aldosteronism, low‐salt diets ameliorate primary aldosteronism by reducing dietary sodium intake, suppressing aldosterone secretion, and enhancing RAAS blockade, thereby alleviating HTA and improving depressive symptoms through central mineralocorticoid modulation [262, 263]. Collectively, among the approaches reviewed, only GLP‐1 receptor agonism (supported by a meta‐analysis of RCTs) and aldosterone‐targeted strategies (supported by clinical cohort and single‐arm trial data) have accumulated human‐level evidence; the remaining candidates require rigorous randomized trials in comorbid populations before they can be considered clinically actionable.

## 8. Conclusion

MD and CD exhibit extensive comorbidity, with their interactions being governed by intricate biological mechanisms. Cardiometabolic disorders, particularly MetS and its associated conditions such as T2D, HTA, and fatty liver disease, drive major depression pathogenesis and progression through pathways including chronic inflammation, IR, and neuroendocrine dysregulation. Conversely, MD, especially depression and anxiety, exacerbate CD deterioration by influencing lifestyle factors and amplifying chronic stress, forming a bidirectional vicious cycle. Despite significant advances in elucidating these mechanisms, critical questions remain unresolved. To move beyond a catalog of associations, the pathways reviewed here can be ranked by the strength of their supporting evidence. HPA‐axis dysfunction and IR are the best‐substantiated shared mechanisms, backed by consistent clinical data and, for the depression–T2D relationship, by bidirectional Mendelian randomization [24]. Gut–brain axis and neuroinflammatory signaling constitute a second tier supported largely by translational and animal models with emerging clinical correlates, whereas disease‐specific pathways such as PPARγ/β‐catenin signaling in psoriasis and CD19‐directed B‐cell modulation remain preclinical and hypothesis‐generating. Prioritizing mechanisms that are both shared across conditions and amenable to existing interventions, most notably GLP‐1‐based and HPA‐targeted strategies, offers the most actionable route from biology to treatment. Future research must clarify the causal relationships between CD and MD and develop novel therapeutic strategies to disrupt this vicious cycle. Combinatorial interventions targeting both disease categories may offer more effective clinical solutions, improving patient quality of life. Thus, investigating MD–CD comorbidity mechanisms remains a critical frontier in medical research.

## Author Contributions

**Hongzhen Du and Gangqiang Du**: conceptualization, writing – original draft, visualization. **Qian Zhang, Yixuan Zhao, Jilin Fan, Yufeng Zhou, and Zihao Sun**: data curation, formal analysis. **Chen Li and Wei Li**: supervision, writing – review and editing.

## Acknowledgments

The authors have nothing to report.

## Funding

This work was supported by the Shandong Provincial Natural Science Foundation (Grants ZR2022MH063 to Wei Li and ZR2022YQ65 and ZR2021MH073 to Chen Li), the Shandong Province Clinical Key Specialty for Department of Rehabilitation Medicine (Grant SLCZDZK‐ZY0101 to Wei Li), the National Natural Science Foundation of China (Grants 82171521 and 82371539 to Chen Li), the Special Funds of the Taishan Scholars Project of Shandong Province (Grant tsqn202211368 to Chen Li), the Shandong Provincial Key Medical and Health Laboratory of Research in Neuropsychiatric Disorders (Binzhou Medical University Hospital), and the Shandong Key Laboratory of Mental Disorders and Intelligent Control.

## Disclosure

No financial or personal relationships influenced the research, analysis, or conclusions presented in this manuscript.

## Conflicts of Interest

The authors declare no conflicts of interest.

## Data Availability

Data sharing is not applicable to this article, as no datasets were generated or analyzed during the current study.
